# Does the Porter formula hold its promise? A weight estimation formula for macrosomic fetuses put to the test

**DOI:** 10.1007/s00404-019-05410-7

**Published:** 2019-12-27

**Authors:** Christoph Weiss, Sabine Enengl, Simon Hermann Enzelsberger, Richard Bernhard Mayer, Peter Oppelt

**Affiliations:** Department of Gynecology, Obstetrics and Gynecological Endocrinology, Kepler University Hospital, Johannes Kepler University Linz, Altenberger Strasse 69, Krankenhausstrasse 26-30, 4040 Linz, Austria

**Keywords:** Macrosomia, Ultrasonography, Fetal weight estimation, Formula, Porter, Hadlock

## Abstract

**Purpose:**

Estimating fetal weight using ultrasound measurements is an essential task in obstetrics departments. Most of the commonly used weight estimation formulas underestimate fetal weight when the actual birthweight exceeds 4000 g. Porter et al. published a specially designed formula in an attempt to improve detection rates for such macrosomic infants. In this study, we question the usefulness of the Porter formula in clinical practice and draw attention to some critical issues concerning the derivation of specialized formulas of this type.

**Methods:**

A retrospective cohort study was carried out, including 4654 singleton pregnancies with a birthweight ≥ 3500 g, with ultrasound examinations performed within 14 days before delivery. Fetal weight estimations derived using the Porter and Hadlock formulas were compared.

**Results:**

Of the macrosomic infants, 27.08% were identified by the Hadlock formula, with a false-positive rate of 4.60%. All macrosomic fetuses were detected using the Porter formula, with a false-positive rate of 100%; 99.96% of all weight estimations using the Porter formula fell within a range of 4300 g ± 10%. The Porter formula only provides macrosomic estimates.

**Conclusions:**

The Porter formula does not succeed in distinguishing macrosomic from normal-weight fetuses. High-risk fetuses with a birthweight ≥ 4500 g in particular are not detected more precisely than with the Hadlock formula. For these reasons, we believe that the Porter formula should not be used in clinical practice. Newly derived weight estimation formulas for macrosomic fetuses must not be based solely on a macrosomic data set.

**Electronic supplementary material:**

The online version of this article (10.1007/s00404-019-05410-7) contains supplementary material, which is available to authorized users.

## Introduction

An important task for obstetricians is to identify disproportionately heavy fetuses—known as macrosomic fetuses—during prenatal visits. Regrettably, the literature does not provide a consistent definition of macrosomia [1]. In general, fetuses with a birthweight (BW) ≥ 4000 g are considered macrosomic. Alternatively, the 95th or 97th percentile, or a BW of ≥ 4500 g, is used for definition [2]. However, there is no doubt that there is a clear association between birth-related fetal injury (e.g., asphyxia or shoulder dystocia) and fetal BW [3–5]. In particular, fetuses weighing more than 4500 g are associated with a significantly increased risk of trauma [1, 6].

The greatest challenge in everyday practice is to detect macrosomic fetuses with sufficient certainty to provide suitable counseling for the expectant parents. Weight estimation formulas based on sonographic fetal measurements have long been in use for this purpose [7]. One of the internationally best-established sets of fetal weight estimation formulas is that by Hadlock et al., published in 1985 [8]. A major disadvantage of almost all formulas in use is undoubtedly that they underestimate the weight of macrosomic fetuses [7, 9]. Over the years, a myriad of weight estimation formulas has been published in the hope of providing the obstetrician with a better tool for fetal weight estimation. Virtually, every measurable fetal variable has already been incorporated into this type of estimation formula, whether with two-dimensional [10] or three-dimensional [11, 12] measurements.

Some authors have argued that specialized formulas for certain weight ranges need to be developed to increase the accuracy of fetal weight estimation [13]. Porter et al. [14] dedicated themselves to the task and published a fetal weight estimation formula for macrosomic fetuses in 2015. They used standardized fetal measurements in a linear regression model to derive their formula. When comparing their new formula with the Hadlock formula, Porter et al. found that it provided a significantly higher detection rate for macrosomic fetuses in their study group.

The aim of the present study was to investigate and question the usefulness of the Porter formula in clinical practice in a large obstetric department for an unselected population. The study also draws attention to some critical issues concerning the derivation of this type of specialized formula and the potentially fatal consequences.

## Methods

### Data collection

This single-center retrospective cohort study included all births between January 1, 2013 and December 31, 2017 at the Department of Gynecology, Obstetrics, and Gynecological Endocrinology at Kepler University, Linz, Austria—the largest obstetrics ward in Austria, with around 3800 births per year. The data were obtained from our in-house computer database of perinatal records.

The inclusion criteria were: singleton pregnancy, liveborn infants with a BW ≥ 3500 g, a complete data set of ultrasound examinations, and—based on the inclusion criteria described by Porter et al. [14]—fetal measurements that had been performed within 14 days before delivery. Only the most recent estimated fetal weight (EFW) was taken into account. The data represent an unselected cross section of the population.

A cut-off value of 3500 g was selected for two reasons: first, the Porter formula was specifically designed to detect macrosomic fetuses when “macrosomia is suspected.” All fetuses in this analysis that had a birthweight well into the normal range were, therefore, excluded. Second, commonly used 2D fetal weight estimation formulas are relatively accurate up to 3500 g [15].

Ultrasound examinations form part of routine prenatal management in the department. The examinations were performed transabdominally by experienced physicians, using high-quality ultrasound systems (Voluson E6 and E8, GE Medical Systems, Zipf, Austria). Women with a normal pregnancy history visit the department for the first time at around 37 + 0 weeks of gestation. A final routine weight estimation is performed at term. In our department, induction of labor is recommended at 40 gestational weeks plus 10 days.

Routine weight estimation included measurements of the biparietal diameter (BPD), head circumference (HC), abdominal circumference (AC), and femur length (FL) in accordance with the International Society of Ultrasound in Obstetrics and Gynecology (ISUOG) recommendations [16]. The Hadlock formula using all four fetal parameters (BPD, HC, AC, and FL) [8] is used for weight estimation in our department and was used in this study.

Gestational age was assessed relative to the crown–rump length measured at the first-trimester ultrasound examination. These measurements were made by physicians in private practice during the mandatory maternal examination. If the crown–rump length was not known, the first day of the last menstrual period was used for gestational age assessment. Delivery data such as BW and sex were filed by midwives in a separate database.

For this study, the EFW provided by the Hadlock formula was compared with the EFW determined by the Porter formula using the same fetal measurements.

### Ethical approval

In accordance with the guidelines, and with confirmation in a written statement by the chairman of the Research Ethics Committee of Upper Austria, no specific ethical approval was necessary for this retrospective study. A waiver of consent was approved.

### Statistics

The accuracy of the two formulas for predicting fetal macrosomia (defined as BW ≥ 4000 g) was tested using absolute error: (|EFW − BW|), percentage error: (EFW − BW/BW × 100), absolute percentage error: (|EFW − BW|/BW × 100), sensitivity, specificity, false-positive rate, false-negative rate, positive predictive value, negative predictive value, and overall accuracy defined as (true-positive plus true-negative)/all cases.

Percentages of EFWs falling within the ± 5% and ± 10% ranges of the actual BW were calculated for both formulas.

On the basis of the findings reported by Faschingbauer et al. [17], a subgroup analysis of all births in the study group within a scan-to-delivery interval of 3 days was performed. Statistical analysis was carried out using the R statistical software package [18]. For numerical variables, *t* tests were carried out. For all analyses, *P* < 0.05 was considered statistically significant.

## Results

A total of 4654 births met the inclusion criteria; 1156 infants (24.84%) had a BW ≥ 4000 g, 110 (2.36%) weighed ≥ 4500 g, and only seven infants weighed more than 5000 g. Table 1 lists the demographic and obstetric characteristics of the study group.

**Table 1:** Demographic and clinical parameters in the study population (*n* = 4654), given as means (± SD)

Maternal age (years)	30.43 (± 5.2)
Gestational age at delivery (days)	280.59 (± 7.3)
Time from fetal weight estimation to delivery (days)	6.00 (± 3.9)
Birth weight (g)	3840.25 (± 268.4)
Gender (male/female)	2741/1913

Among the macrosomic infants (BW ≥ 4000 g), 313 (27.08%) were identified using the Hadlock formula, with a false-positive rate of 4.60%. In contrast, all macrosomic fetuses were detected using the Porter formula, with a false-positive rate of 100%. Ten (9%) of the infants weighing more than 4500 g were correctly detected with the Hadlock formula, the same number as with the Porter formula. None of the infants weighing ≥ 5000 g was detected by either formula. The mean time interval between measurement and birth in infants ≥ 4000 g was 5.8 days (± SD 4.0 days). In general, the mean interval between ultrasound examination and birth was 6.0 days (± SD 3.9 days, *P* = 0.18).

The individual classification parameters for each formula are summarized in Table 2. Table 3 shows the mean percentage error, mean absolute percentage error, and mean absolute error for both formulas.

**Table 2:** Classification parameters for macrosomia with each formula

	Hadlock (%)	Porter (%)
Sensitivity	27.08	100
Specificity	95.40	0
PPV	66.03	24.83
NPV	79.83	0
FNR	72.92	0
FPR	4.60	100
Overall accuracy	78.43	24.83

*FNR* false-negative rate, *FPR* false-positive rate, *NPV* negative predictive value, *PPV* positive predictive value

**Table 3:** Mean percentage error, mean absolute percentage error, and mean absolute error values derived from the Hadlock and Porter formulas for all births in the study group and classified into birth weights < 4000 g and ≥ 4000 g

	Hadlock	Porter	*P* value
BW ≥ 3500 g (*n* = 4654)			
Mean PE (± SD)	− 6.78% (± 7.7%)	11.51% (± 7.0%)	< 0.0001
Mean APE (± SD)	8.42% (± 5.8%)	12.03% (± 6.1%)	< 0.0001
Mean AE (± SD)	327.15 g (± 236.6 g)	447.58 g (± 209.5 g)	< 0.0001
BW < 4000 g (*n* = 3498)			
Mean PE (± SD)	− 5.95 (± 7.5%)	14.65% (± 4.4%)	< 0.0001
Mean APE (± SD)	7.82 (± 5.5%)	14.65% (± 4.4%)	< 0.0001
Mean AE (± SD)	291.28 g (± 196.7 g)	538.41 g (± 143.8 g)	< 0.0001
BW ≥ 4000 g (*n* = 1156)			
Mean PE (± SD)	− 9.32% (± 7.8%)	2.01% (± 4.4%)	< 0.0001
Mean APE (± SD)	10.26% (± 6.5%)	4.09% (± 2.6%)	< 0.0001
Mean AE (± SD)	435.72 g (± 284.4 g)	172.72 g (± 116.2 g)	< 0.0001

*AE* absolute error, *APE* absolute percentage error, *BW* birth weight, *PE* percentage error

**Fig. 1:**
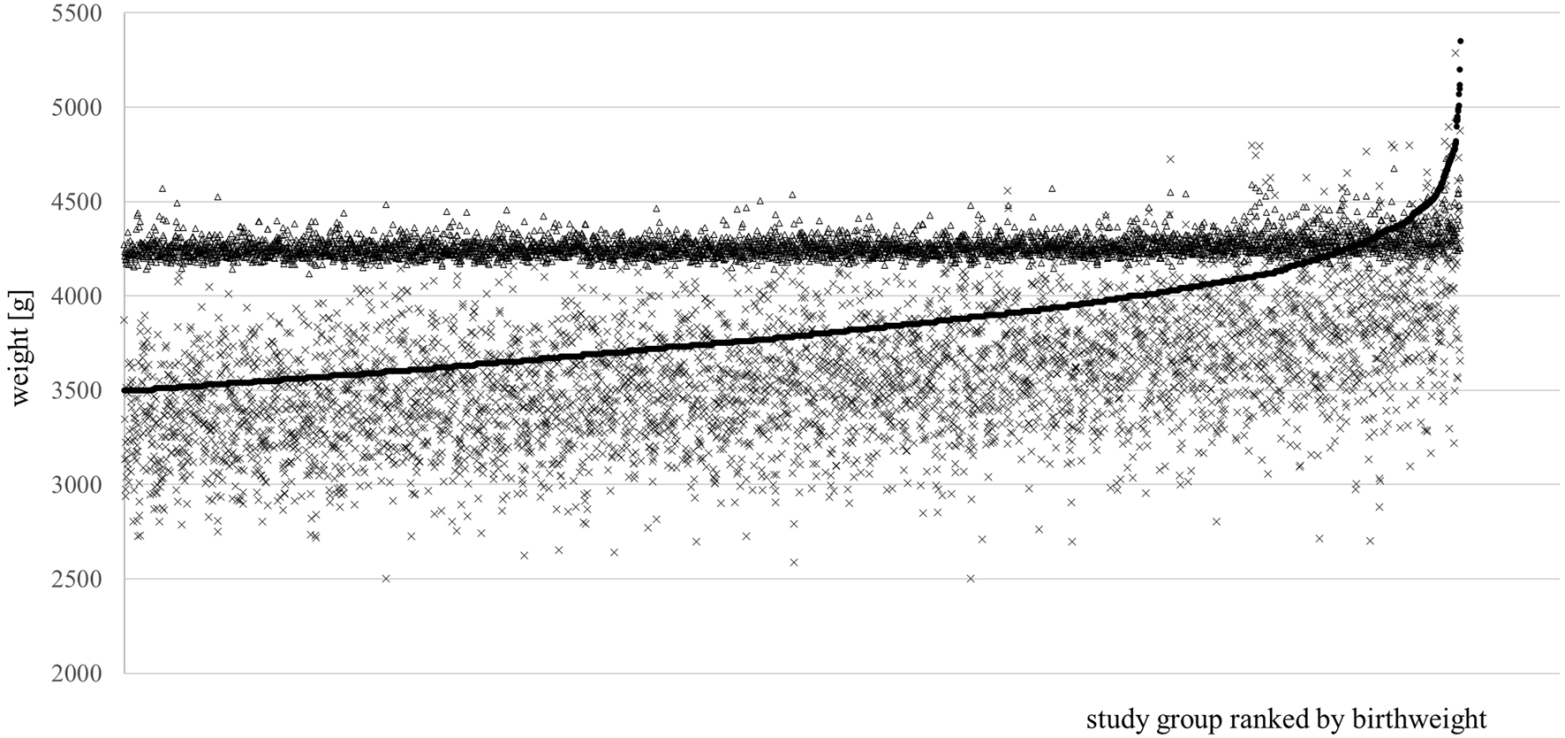
Scatter plot of all births (*n* = 4654), ranked by increasing birthweight (BW). In all, 313 (27.08%) of the macrosomic infants (BW ≥ 4000 g) were correctly identified using the Hadlock formula; 1156 (100%) were detected using the Porter formula. The favorable appearance is achieved by the fact that the Porter formula sets the estimated weights within a very narrow band at around 4300 g; 99.44% of all weight estimations using the Porter formula were within a range of 4300 g ± 5%, leading to massive overestimation of normal-weight fetuses. •, Actual birthweight; *∆*, fetal weight estimated with the Porter formula; *x*, fetal weight estimated with the Hadlock formula

The overall percentages of weight estimates falling within ± 5% and ± 10% of the BW using the Hadlock formula were 33.15% and 64.42%, respectively; the corresponding figures for the Porter formula were 15.58% and 38.09%. For macrosomic infants, 22.66% of the Hadlock estimates and 63.67% of the Porter estimates were within ± 5% of the BW; 51.99% (Hadlock) and 97.75% (Porter) were within ± 10%. In other words, in the study group as a whole, 99.44% of all weight estimations using the Porter formula were within a range of 4300 g ± 5%. and 99.96% were within a range of 4300 g ± 10%, with a mean estimated weight of 4263 g (SD ± 56.78 g) (Fig.1).

In the subgroup analysis including only births of macrosomic fetuses with a weight estimation within 3 days of delivery (*n* = 1388), the Hadlock formula showed improved sensitivity in comparison with the whole-study group—50.25% and 27.08%, respectively. However, the Porter formula did not show any improved performance. Table 4 lists the demographic and obstetric characteristics in this subgroup. The classification parameters are summarized in Table 5.

**Table 4:** Demographic and clinical parameters for the subgroup analysis, including only births with a fetal weight estimation ≤ 3 days before delivery (*n* = 1388), given as means (± SD)

Maternal age (years)	30.34 (± 5.3)
Gestational age at delivery (days)	279.21 (± 7.2)
Time from fetal weight estimation to delivery (days)	1.44 (± 0.8)
Birth weight (g)	3863.27 (± 281.0)
Gender (male/female)	802/586

**Table 5:** Classification parameters for macrosomia with each formula in the subgroup analysis, including only births with a fetal weight estimation ≤ 3 days before delivery (*n* = 1388)

	Hadlock (%)	Porter (%)
Sensitivity	50.25	100
Specificity	88.65	0
PPV	63.55	28.24
NPV	81.91	0
FNR	49.74	0
FPR	11.35	100
Overall accuracy	77.81	28.24

*FNR* false-negative rate, *FPR* false-positive rate, *NPV* negative predictive value, *PPV* positive predictive value

## Discussion

Although a vast number of weight estimation formulas for macrosomic fetuses have been published in the past, many more will undoubtedly follow. This is not surprising, as we are still failing to detect macrosomic fetuses with satisfactory accuracy. Obstetricians worldwide are still using weight estimation formulas developed in the 1980s, such as the Hadlock formulas, or those of Merz [19] and Shepard [20], published in 1988 and 1982, respectively. Those formulas, which were never intended specifically for large fetuses but rather represent “all-rounder” formulas, notoriously underestimate the weight of macrosomic fetuses [7, 9]. They all fail to detect such at-risk fetuses with sufficient certainty. Nevertheless, the Hadlock formulas still show favorable results in comparison with others [21].

Porter et al. [14] stated that they were able to diagnose macrosomic fetuses significantly better with their new formula than with the well-established Hadlock formula. Superficially, the figures they present seem to be convincing. However, if one takes a closer look at the “behavior” of the Porter formula, one quickly realizes that the apparently good hit ratio for fetuses with a BW ≥ 4000 g is achieved, only because the Porter formula puts virtually all weight estimates within a very narrow band around 4300 g. This behavior is best explained by the selection criteria used for the formula-finding group. The formula is based on a training data set (*n* = 201) including only fetuses with a BW ≥ 4000 g. If this formula is tested with a data set that similarly includes only fetuses with a BW ≥ 4000 g, naturally excellent results are produced. The mean BW for their whole-study group was 4236 g. Moreover, Porter et al. included the data set of the formula-finding group in the comparison between their new formula and the Hadlock formula. This procedure appears at least questionable. They never applied their formula to fetuses with a BW below 4000 g.

With this mode of derivation, such a formula will never succeed in distinguishing macrosomic fetuses from normal-weight ones. It will always deliver a macrosomic fetal weight estimation. Bluntly put, no matter what measurements you enter, the Porter formula always delivers a value of around 4300 g. This also applies to children with a BW well below 3500 g. In fetuses with a BW between 1014 g and 5350 g, the lowest weight estimate produced by the Porter formula was 4105 g (see supplementary data).

Furthermore, Porter et al. [14] did not define any rules or cut-off values for when their formula should be applied. They only vaguely stated that the formula “should be considered when macrosomia is suspected.” Used in this way, the Porter formula will always confirm such a suspicion, even when the real fetal weight is far below 4000 g (see supplementary data).

This has serious clinical consequences. On one hand, normal-weight fetuses are massively overestimated, and on the other hand, fetuses with the highest birth risk (BW > 4500 g) will not be detected. The same problem applies to the formula for macrosomic fetuses published by Hart et al. in 2010 [22, 23].

If such formulas were to be implemented in everyday clinical practice, their unreliable results would lead to completely unnecessary uncertainty on the part of expectant mothers and obstetricians alike. Many women with normal-weight fetuses would unnecessarily be classified as having pathological findings. Using the Porter formula, virtually, all pregnant women would be diagnosed as having macrosomic fetuses.

Faschingbauer et al. [17] showed that the optimal scan-to-delivery interval for detecting macrosomic fetuses is 3 days. Even when these findings are taken into account, the Porter formula does not show better parameters.

It has been shown previously that labor abnormalities such as arrested labor are more likely to be diagnosed when fetal macrosomia is suspected despite the real BW [24, 25]. The same applies to cesarean delivery rates. Melamed et al. showed that cesarean delivery rates increase by up to 2.5 times when the fetal weight estimate is ≥ 4000 g regardless of the actual BW [26]. Just recently, similar results were published by Vitner et al., showing an increase even up to 3.5 times [27]. Blackwell et al. calculated that overestimation of fetal weight on ultrasound lowers the threshold for cesarean delivery for labor arrest even when the EFW is < 4000 g [28].

Predicted fetal macrosomia is also associated with a higher rate of labor induction [27, 29], even though there are no recommendations on this topic. On the contrary, several authors have argued that suspected macrosomia alone does not justify induction of labor or primary cesarean delivery [30–32]. The American College of Obstetricians and Gynecologists (ACOG) clearly states that suspected fetal macrosomia is not an indication for inducing labor [33].

In the past, virtually every measurable fetal and/or maternal variable has been used for formula derivation by linear regression. Alas, no particular formula emerged showing a sufficient accuracy in detecting macrosomic fetuses. Thus, the authors of this study strongly believe that no additional fetal weight estimation formulas derived by linear regression are needed. As Kehl et al. already stated in 2012 [34], “weight estimation with conventional biometric parameters by 2D ultrasound has reached its limits”. To use the existing formulas more effectively, some authors have favored a “two-step procedure” for fetal weight estimation [35]. In such an approach, in a first step, the weight range is delineated by one or more sonographic parameters (e.g., AC). Depending on the results, in a second step, fetal weight is estimated by a formula selected by defined thresholds [36, 37]. Whether such an approach enables the obstetrician in clinical practice to improve fetal and/or maternal outcome still has to be shown through clinical trials. Because, not everything that appears to be significant in a published report proves to be of clinical relevance. Perhaps using completely new approaches, e.g., machine learning algorithms, we will succeed in detecting fetuses at risk with sufficient accuracy [38]. The future will tell.

For the present, a macrosomic fetus in utero will continue to present a diagnostic dilemma. All too easily, today’s highly sophisticated ultrasound equipment may lead to the misjudgment that we are able to estimate fetal weight better than is actually the case. The current ACOG Practice Bulletin reflects this by stating rather mischievously that “an accurate diagnosis of macrosomia can only be made by weighing the newborn after delivery” [33].

### Strengths and limitations of the study

The retrospective character and the single-center setting in this study can certainly be regarded as limiting factors. In our opinion, these weaknesses are compensated for by the size of the study group. It might be argued that including fetal measurements up to 14 days before delivery might lead to further distortion of the fetal weight estimates. As stated above, this time interval was chosen on the basis of the inclusion criteria used by Porter et al. [14]. In any case, the mean interval between ultrasound examinations and birth was 6 days for the whole-study group. In our opinion, this resembles a real-life situation.

We were not able to provide demographic parameters concerning preexisting diabetes mellitus, gestational diabetes, or the women’s body mass index. This is because we would have had to obtain such data by a manual review of all clinical reports.

## Conclusion

In our opinion, the Porter formula does not offer any clinical benefit in detecting macrosomic fetuses. Using the Porter formula results in a massive overestimation of normal-weight fetuses. This would lead to an unnecessary increase in rates of labor induction and cesarean sections. Furthermore, high-risk fetuses with a BW ≥ 4500 g in particular are not detected more precisely than with the Hadlock formula. For these reasons, we believe that the Porter formula should not be used in clinical practice.

Newly derived weight estimation formulas for macrosomic fetuses must not be based solely on a macrosomic data set. If they are, such a formula will never be capable of distinguishing between macrosomic and normal-weight fetuses.

## Electronic supplementary material

Below is the link to the electronic supplementary material.

Supplementary material 1 (DOCX 23 kb)

Supplementary material 2 (DOCX 14 kb)
