# Supplementary material for: Does the Porter formula hold its promise? A weight estimation formula for macrosomic fetuses put to the test
Source: Arch Gynecol Obstet. 2019 Dec 27;301(1):129–35. doi: 10.1007/s00404-019-05410-7 (PMC7028832; doi:10.1007/s00404-019-05410-7)
Supplement: Supplementary file 1 — Supplementary material 1 (DOCX 23 kb) [file 404_2019_5410_MOESM1_ESM.docx]

# Supplement

To provide an even more spectacular illustration of the striking behavior of the Porter formula — delivering a value of around 4300 g no matter what fetal measurements are entered — we performed a separate analysis including birth weights (BWs) far below 3500 g. Apart from the BW, the inclusion criteria were the same as in the original study group. The lowest BW in this group was 1014 g, and the lowest weight estimate produced by the Porter formula was 4105 g. Of course, applying the Porter formula to such small fetuses was never intended by Porter el al., who stated very precisely that their formula should only be considered “when macrosomia is suspected.”

The demographic and clinical parameters of the “whole population” dataset (*n* = 11152) are given below in Supplementary Table 1.

Percentages of estimated fetal weights falling within the ± 5% and ± 10% ranges of the actual BW were calculated for both formulas for this extended dataset. For all infants in this extended study group, 40.41% of the Hadlock estimates and 6.62% of the Porter estimates were within ± 5% of the BW; 71.71% (Hadlock) and 15.90% (Porter) were within ± 10%.

Based on the saying, “a picture is worth a thousand words,” the quintessence of the shortcomings of the Porter formula are shown in Supplementary Fig. 1.

**Legend**

**Supplementary Fig. 1**Scatter plot of all births (n = 11152), ranked by increasing birthweight (BW). The favorable appearance is achieved by the fact that the Porter formula sets the estimated weights within a very narrow band at around 4300 g;

•, Actual birthweight; ∆, fetal weight estimated with the Porter formula; x, fetal weight estimated with the Hadlock formula
