# Supplementary material for: Does the Porter formula hold its promise? A weight estimation formula for macrosomic fetuses put to the test
Source: Arch Gynecol Obstet. 2019 Dec 27;301(1):129–35. doi: 10.1007/s00404-019-05410-7 (PMC7028832; doi:10.1007/s00404-019-05410-7)
Supplement: Supplementary file 2 — Supplementary material 2 (DOCX 14 kb) [file 404_2019_5410_MOESM2_ESM.docx]

**Supplementary Table 1**Demographic and clinical parameters in the study population (*n* = 11152), given as means (± SD)

| Maternal age (years) | 30.37 (± 5.4) |
| --- | --- |
| Gestational age at delivery (days) | 276.71 (± 9.8) |
| Time from fetal weight estimation to delivery (days) | 5.69 (± 4.0) |
| Birth weight (g) | 3386.11 (± 496.5) |
| Gender (male/female) | 5773 / 5379 |
